# Allogamy-Autogamy Switch Enhance Assortative Mating in the Allotetraploid *Centaurea seridis* L. Coexisting with the Diploid *Centaurea aspera* L. and Triggers the Asymmetrical Formation of Triploid Hybrids

**DOI:** 10.1371/journal.pone.0140465

**Published:** 2015-10-15

**Authors:** María Ferriol, Alfonso Garmendia, Ana Gonzalez, Hugo Merle

**Affiliations:** 1 Instituto Agroforestal Mediterráneo, Universitat Politècnica de València, Valencia, Spain; 2 Departamento de Ecosistemas Agroforestales, Universitat Politècnica de València, Valencia, Spain; Università di Pisa, ITALY

## Abstract

Hybridization between tetraploids and their related diploids is generally unsuccessful in *Centaurea*, hence natural formation of triploid hybrids is rare. In contrast, the diploid *Centaurea aspera* and the allotetraploid *C*. *seridis* coexist in several contact zones where a high frequency of triploid hybrids is found. We analyzed the floral biology of the three taxa to identify reproductive isolation mechanisms that allow their coexistence. Flowering phenology was recorded, and controlled pollinations within and between the three taxa were performed in the field. Ploidy level and germination of progeny were also assessed. There was a 50% flowering overlap which indicated a phenological shift. Diploids were strictly allogamous and did not display mentor effects, while tetraploids were found to be highly autogamous. This breakdown of self-incompatibility by polyploids is first described in *Centaurea*. The asymmetrical formation of the hybrid was also found: all the triploid intact cypselae came from the diploid mothers pollinated by the pollen of tetraploids. Pollen and eggs from triploids were totally sterile, acting as a strong triploid block. These prezygotic isolation mechanisms ensured higher assortative mating in tetraploids than in diploids, improving their persistence in the contact zones. However these mechanisms can also be the cause of the low genetic diversity and high genetic structure observed in *C*. *seridis*.

## Introduction

Nowadays there is no doubt about the central role of polyploidization in the evolution of flowering plants [1]. While there is evidence for ancestral whole genome duplications in extant angiosperms, nearly one third are recent polyploids, and 15% of speciation events are estimated to result from polyploidy [2].

*Centaurea* L. (Asteraceae) is a recently evolved genus that presents a high diversification and speciation rate due to the existence of cycles of polyploidy and descending dysploidy, as well as hybridization events [3, 4]. These evolutionary dynamics have led to the existence of several contact zones where diploid and both auto- and allo-polyploid individuals coexist [5–10]. In most of these contact zones, particularly those described for the subgenera *Jacea* (Mill.) Hayek and *Acrolophus* (Cass.) Dobrocz., there are strong reproductive barriers that prevent effective hybridization between diploids and tetraploids. Therefore in contrast to homoploid crosses, individuals with different ploidy levels rarely hybridize and when this occurs, hybridization results in tetraploid hybrids rather than triploids [5, 7, 8, 11]. In *Centaurea*, both pre-zygotic and post-zygotic barriers are causing this reproductive isolation. Pre-zygotic barriers include spatial segregation, which leads to parapatric distributions with very few and small contact zones [12, 13]; microhabitat segregation in contact zones [9]; a certain phenological shift [7]; and mentor effects (induction of selfing in self-incompatible species by mixed loads of self and heterospecific pollen, which may have a different ploidy level) [6]. In other angiosperms, a breakdown of self-incompatibility in tetraploids has been described but not to date in *Centaurea*. On the other hand, post-zygotic mechanisms also act through a lack of germination, a low viability, or a high degree of sterility of the rarely formed triploid hybrids [9, 10].

Conversely in the subgenus *Seridia* (Juss.) Czerep., widely distributed diploid *C*. *aspera* L. and allotetraploid *C*. *seridis* L. hybridize frequently and lead to triploid hybrids *C*. × *subdecurrens* Pau, which have also been found to be sterile [14, 15]. These three taxa are closely related. In a previous genetic analysis using microsatellites, 56% of the loci showed fixed or nearly fixed heterozygosity in *C*. *seridis*, and 77% of its alleles were shared with *C*. *aspera* [15]. These results supported that *C*. *seridis* is an allotetraploid, being *C*. *aspera* one of its parental taxa. Genetic analyses supported that *C*. × *subdecurrens* is a true F1 hybrid and that backcrossing events and gene flow are very rare, or even absent [14, 15]. Microsatellite markers also showed that *C*. *seridis* has a very low genetic diversity, with sampled individuals grouped in two highly differentiated populations [15].

Both taxa are distributed in the western Mediterranean. *Centaurea seridis* mainly grows on coastal sand and pebble dunes in the Spanish central and southern Mediterranean coast and Moroccan northern coast [16]. *Centaurea aspera* has a broader distribution area, growing inland and on fixed and semi-fixed coastal dunes in the western Mediterranean part including countries such as Italy, France, Spain and Morocco among others [17]. The distribution area of *C*. *seridis* is surrounded by the distribution area of *C*. *aspera*. In Spain, both taxa grow in sympatry when dune habitats include mobile, semi-fixed and fixed dunes. Consequently, whereas the distribution area of *C*. *aspera* has relatively low overlapping with that of *C*. *seridis*, the reciprocal is not true, and a high portion of *C*. *seridis* distribution area overlaps with that of *C*. *aspera*. Accordingly, several extensive contact zones have been recorded along the Spanish and Moroccan coast [16, 18]. The origin of these contact zones is not obvious. They might be primary contact zones, in which allopolyploids established, and spread along the coast, as secondary hybridization involving allopolyploids and their diploid parents is not common in nature [19]. However, we cannot rule out that the contact zones between *C*. *aspera* and *C*. *seridis* are secondary, as they are often found in highly disturbed coastal dunes, where parental species distribution may be altered and novel or open environments in which hybrids are able to establish themselves may be provided [14, 20–22].

In this study, controlled crosses were carried out within and between the three taxa involved in the contact zone of El Saler [14], and progeny ploidy and germination capacity were examined. We aimed to analyze the possible reproductive isolation between diploids and tetraploids, which leads to the well-established coexistence of the three taxa in the contact zones. The questions we posed were: (i) although flowering periods widely overlap [23], does some phenological shift exist that could act as a pre-zygotic barrier between diploids and tetraploids to some extent?; (ii) are triploid eggs and pollen totally sterile or can they act as a triploid bridge to some degree? Although genetic analyses have shown that the gene flow between diploids and tetraploid is rare or absent, no artificial crosses have been performed to quantify this; (iii) does a shift from allogamy to autogamy occur in the different taxa?; (iv) are there any differences in seed sets between intra- and interspecific crosses?; and (v) what are the ploidy levels of intraspecific offspring and interspecific hybrids? The responses to these questions can allow us to elucidate if asymmetries exist in the formation of triploids by diploid *vs*. tetraploid mothers.

## Materials and Methods

No specific permissions were required for these locations. The field studies did not involve endangered or protected species.

### Phenological traits

The flowering phenology of *C*. *aspera*, *C*. *seridis*, and *C*. × *subdecurrens* was assessed during a 2-year field observation period (2004 and 2005) at the polyploid complex of El Saler (Valencia). The number of capitula at anthesis was recorded once a week from January to December for 36 individuals of *C*. *aspera*, 18 of *C*. *seridis* and 5 of *C*. × *subdecurrens* in 2004; and 19, 12, and 9 individuals, respectively, in 2005. The mean number of capitula at anthesis was calculated weekly and depicted graphically. To compare the flowering curves for diploids and tetraploids, an overlap index was calculated following Husband and Schemske [24].

### Controlled pollinations

Six controlled pollination treatments were performed: (i) hand self-pollination; (ii) cross-pollination between individuals of the same ploidy level (intraploidy): A×A (*C*. *aspera*), S×S (*C*. *seridis*) and H×H (*C*. × *subdecurrens*); (iii) cross-pollination between diploid and tetraploid individuals (interploidy): A×S (mother *C*. *aspera* × father *C*. *seridis*) and S×A (reciprocally); (iv) cross-pollination between diploids and triploids: A×H (mother *C*. *aspera* × father *C*. × *subdecurrens*) and H×A (reciprocally); (v) cross-pollination between triploids and tetraploids: S×H and H×S, and (vi) the bagged treatment.

All the crosses were carried out on individuals that grew naturally in the contact zone of El Saler [14]. Individuals were randomly selected with a randomized GPS coordinate system to obtain at least 15 capitula per treatment and taxon. Treatments were conducted during the flowering period in four consecutive episodes from June to July 2013 (S1 Table). First hand-pollination dates were: June 19^th^ (56 capitula), June 27^th^ (47 capitula), June 29^th^ (80 capitula) and July 8^th^ (59 capitula).

Pollinations were performed with newly open capitula, which were placed inside semi-permeable nylon bags prior to anthesis. Donor capitula were also bagged prior to anthesis. At anthesis, capitula were brushed gently against each other once a day on 2 consecutive days. In the bagged treatment, capitula were only bagged without brushing. This treatment tested the capacity of automatic self-pollination, probably with pollen from the same flower (true self-pollination), but also with some pollen from other flowers of the same capitulum (geitonogamy). In the self-pollinations, each treated capitulum received pollen from two donor capitula of the same plant. Therefore self-pollination treatment included a mix of geitonogamy (possibly the majority) and true self-pollination. In the cross-pollinations, each treated capitulum received pollen from 10–15 donors. The flowers of the treated capitula were not emasculated given the difficulty to handle Asteraceae capitula. Consequently, it was not possible to avoid self-pollination and the resulting cypselae may have originated by xenogamy, geitonogamy, and true self-pollination.

### Progeny analysis

After pollinations, capitula were re-bagged for 5 weeks until fruit set. For each treatment, total cypselae per capitulum were counted, as were those included in the following categories: (i) intact cypselae, when they were plump and had a fully developed embryo; (ii) empty or depredated cypselae; (iii) mature cypselae with small or aborted embryos; (iii) cypselae filled with nonembryonic tissue.

The ploidy level and germination capacity of cypselae with sufficiently developed embryos were analyzed. Cypselae were disinfected with 0.5% NaClO solution for 20 min, washed 3 times for 5 min in distilled water and hydrated in parafilm-closed Petri dishes for 24 h at 20°C. Subsequently, each cypsela was cut at 2/3 from the epicotyl and the pericarp was removed. The 1/3 distal cotyledonary tissue was used to determine the ploidy level of each embryo by flow cytometry, as described in Garmendia *et al*. [16]. The rest of the embryo (2/3) was placed in wet Petri dishes at room temperature and in natural light to analyze germination of cut embryos. This did not represent natural germination capacity of cypselae since pericarp was removed and a cut was made in the cotyledons.

### Statistical analyses

The average, standard error, skew, kurtosis, frequency distribution and density curve of the number of cypselae per capitulum were assessed for each pollination treatment and taxon. The normality and homogeneity of variances were checked with a Shapiro-Wilk test and a Levene test, respectively. To compare the actual distribution with a normal one with the same mean and standard deviation, both were represented using the function “density()” in “R”, which computes a kernel density estimation using a gaussian smoothing (S1 Fig).

Due to the lack of normality, non-parametric methods were selected for the comparison of medians: the median number of cypselae was compared among treatments and repetitions by a Kruskal-Wallis rank sum test [25] and post hoc Dunn's test [26].

Likelihood ratio tests [27] were used to compare the fitness of the models with different variable combinations (treatment, repetition or both). For either taxa, the model using the interaction of both variables was significantly better than those using any variable alone. Akaike Information Criterion (AIC) was also used to compare between parametric (ANOVA) and non parametric models (Poisson model [28] and zero-inflated Poisson model [29]). The last two were the best models for *C*. *aspera* and *C*. *seridis* respectively. In addition, a Vuong test [30] was used to compare models when possible, especially for *C*. *seridis* between Poisson and zero-inflated Poisson models, for which the last one was significantly better than the former (p = 0.00097). Consequently, non-parametric models using the interaction between treatments and repetitions were selected, using the Poisson model for *C*. *aspera* and the zero-inflated Poisson model for *C*. *seridis*. All the analyses were carried out using R [31] with the following extra libraries: dunn.test, for the *post hoc* tests; pscl, for the Vuong test; lmtest, for the likelihood ratio tests; psych, for the descriptive statistics; and class.

## Results

### Phenological traits

**Fig 1:**
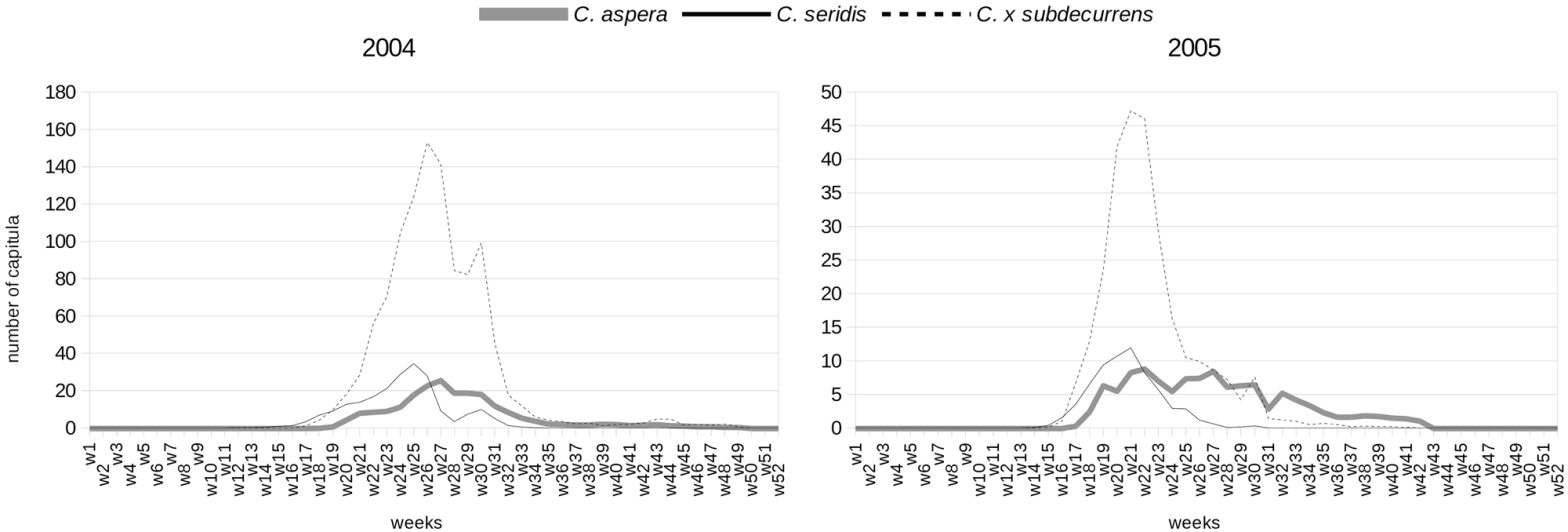
Flowering phenology of the diploid *Centaurea aspera*, the tetraploid *C*. *seridis*, and the triploid hybrid *C*. × *subdecurrens*. w1-w52: weeks of field observations; w1: start at January 5th-11th for 2004 and January 3rd-9th for 2005.

During the 2-year observation period (Fig 1), *Centaurea seridis* always bloomed first and had the shortest flowering period, from the start of April to the start of August (17–21 weeks), with a flowering peak that occurred from late May to mid-June. *Centaurea aspera* was the last to bloom, 2 to 3 weeks later than *C*. *seridis*, and had the longest flowering period, from the start of May to the start of December (27–33 weeks), with a flowering peak that varied from late May to late June. Hybrids displayed an intermediate phenology, with a flowering period that lasted 23–33 weeks (from mid-April to the start of November), and a flowering peak which occurred from late May to late June. The overlap index between *C*. *aspera* and *C*. *seridis* was 55% in 2004 and 44% in 2005.

### Breeding system and reproductive isolation among taxa

Including all the pollination treatments, 863 cypselae were collected from 242 capitula.

#### Self pollinations *vs*. intraploidy cross-pollinations

In *C*. *aspera*, 16 self-pollinations yielded only four cypselae, one of which had a small embryo and three were produced in a single capitulum (0.25 +- 0.19 cypselae per capitulum) (Table 1 and S1 Fig). In contrast, 16 intraploidy cross-pollinations produced 73 cypselae, five of which were depredated and five other showed abnormalities (4.56 +- 1.24 cypselae per capitulum). The average number of cypselae per capitulum of self- and intraploidy pollinations was significantly different (Kruskal-Wallis test: p = 0.0000005, S2 Table). The bagged treatment yielded no cypselae.

**Table 1:** Number of cypselae, category and ploidy level obtained for each pollination treatment and taxon.

Maternal taxon	Type of cross	N	Mean	Se	Skew	Kurt.	Total n° cypselae	Intact	Ploidy	Small	Ploidy	Tiss.	Depr.	Aberr. %
*C*. *aspera*	Selfing	16	0.25	0.19	2.82	6.99	4	3	2*x*(3)	1	-	0	0	25
	A×A	16	4.56	1.24	0.59	-1.00	73	63	2*x*(63)	2	3*x*(1)	3	5	7.4
	A×S	16	3.00	0.68	0.22	-1.48	48	9	3*x* (8)*	14	3x (2)	5	20	67.9
	A×H	16	0.00	0.00	—	—	0	0	-	0	-	0	0	—
	Bagged	17	0.00	0.00	—	—	0	0	-	0	-	0	0	—
*C*. *seridis*	Selfing	16	10.63	2.16	0.08	-1.32	170	153	4*x*(153)	4	4*x*(2). 3*x*(1)	0	13	2.5
	S×S	17	9.76	1.84	0.48	-0.67	166	133	4*x*(133)	13	4*x*(5)*	0	20	8.9
	S×A	16	11.00	2.19	0.03	-1.56	176	140	4*x*(140)	2	4*x*(2)	0	34	1.4
	S×H	17	10.06	1.86	0.14	-1.34	171	135	4*x*(135)	3	4*x*(2). 3*x*(1)	0	33	2.2
	Bagged	17	3.24	1.47	1.93	2.85	55	50	4*x*(50)	5	4*x*(3). 3*x*(1)	0	0	9.1
*C*. *× subdecurrens*	Selfing	15	0.00	0.00	—	—	0	0	-	0	-	0	0	—
	H×H	15	0.00	0.00	—	—	0	0	-	0	-	0	0	—
	H×A	15	0.00	0.00	—	—	0	0	-	0	-	0	0	—
	H×S	16	0.00	0.00	—	—	0	0	-	0	-	0	0	—
	Bagged	17	0.00	0.00	—	—	0	0	-	0	-	0	0	—
Total		242					863	686						

N, number of treated capitula; Se, standard error; Kurt., kurtosis; Intact, cypselae with fully developed embryo; Small, cypselae with small or aborted embryos; Tiss., cypselae filled with nonembryonic tissue; Depr., empty or depredated cypselae; Aberr., percentage of aberrations. In Ploidy, number in parenthesis corresponds to the number of cypsela that has been effectively evaluated.

*One aneuploid.

In *C*. *seridis*, unlike *C*. *aspera*, the average number of cypselae obtained with both pollination treatments (self-pollination and S×S) were similar with nonsignificant differences (S2 Table). Sixteen self pollinations yielded 170 cypselae, 13 of which were depredated or empty and four other had small embryos (10.63 +- 2.16 cypselae per capitulum), while 17 intraploidy crosses yielded 166 cypselae, 20 of which were depredated and 13 had small embryos (9.76 +- 1.84 cypselae per capitulum) (Table 1).

In *C*. *seridis*, 17 bagged pollinations (capitula enclosed with no brushing) resulted in 55 cypselae, five of which had small embryos (3.24 +- 1.47 cypselae per capitulum). This number was significantly lower than that observed in self-pollinations (Kruskal-Wallis test: p = 0.022). The intraploidy crosses in *C*. *seridis* yielded significantly more cypselae per capitulum than those in *C*. *aspera* (Mann-Whitney *U*-test: p < 0.05).

Triploid hybrid *C*. × *subdecurrens* was completely sterile in both the self- and intraploidy pollinations. No cypselae were obtained in the 30 capitula included in both treatments (Table 1). Therefore in relation to the breeding system, diploids were considered self-incompatible, tetraploids were self-compatible, and triploids were sterile.

#### Interploidy pollination

**Fig 2:**
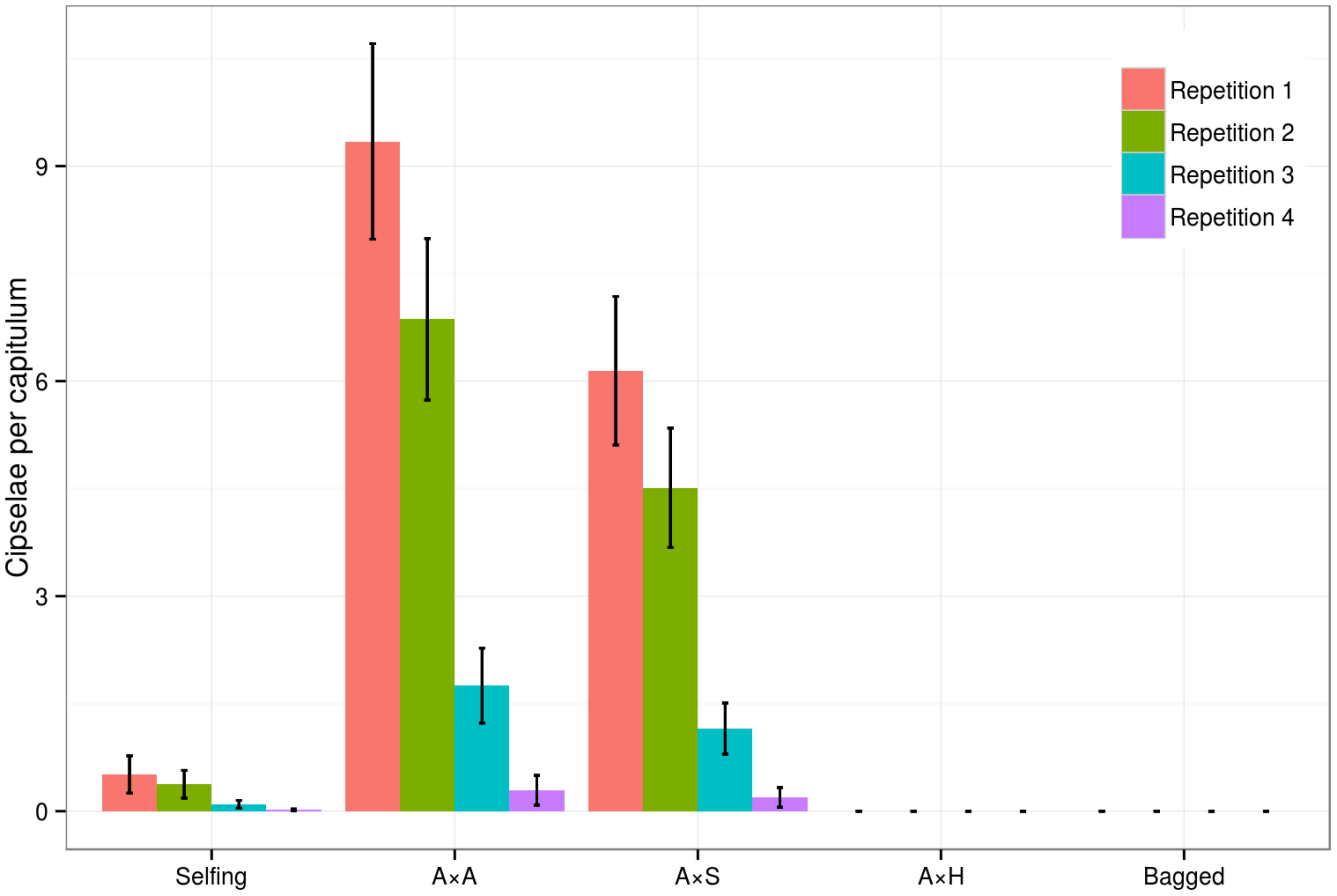
Predicted values for the diploid *Centaurea aspera* by the Poisson model. Colors represent the repetitions; red: first repetition; green: second; blue: third and purple: fourth. Selfing: self-pollination; A×A: cross-pollination between individuals of *C*. *aspera* (intraploidy); A×S: coss-pollination between mother diploid *C*. *aspera* and father tetraploid *C*. *seridis*; A×H: cross-pollination between mother diploid *C*. *aspera* and father triploid *C*. × *subdecurrens*; bagged: bagged treatment without brushing. Error bars indicate the standard error for the Poisson model estimates.

The pollen of *C*. *seridis* could fertilize the ovules of the self-incompatible *C*. *aspera* (A×S), and 48 cypselae were produced in 16 capitula (Table 1 and Fig 2). However, there were very few intact cypselae (9) and many depredated or empty cypselae (20), cypselae with small embryos (14), and cypselae with nonembryonic tissue (5) (Table 1). There were fewer cypselae in the A×S treatment than in the intraploidy pollination of *C*. *aspera* (3.00 +- 0.68 and 4.56 +- 1.24, respectively; Fig 2), but no significant differences were found (S2 Table). However, the percentage of abnormal embryos was considerably higher in A×S than in A×A (67.9% and 7.4%, respectively) (Table 1). The pollen of *C*. × *subdecurrens* was infertile when applied to the *C*. *aspera* capitula, and 16 interploidy pollinations yielded no cypselae.

**Fig 3:**
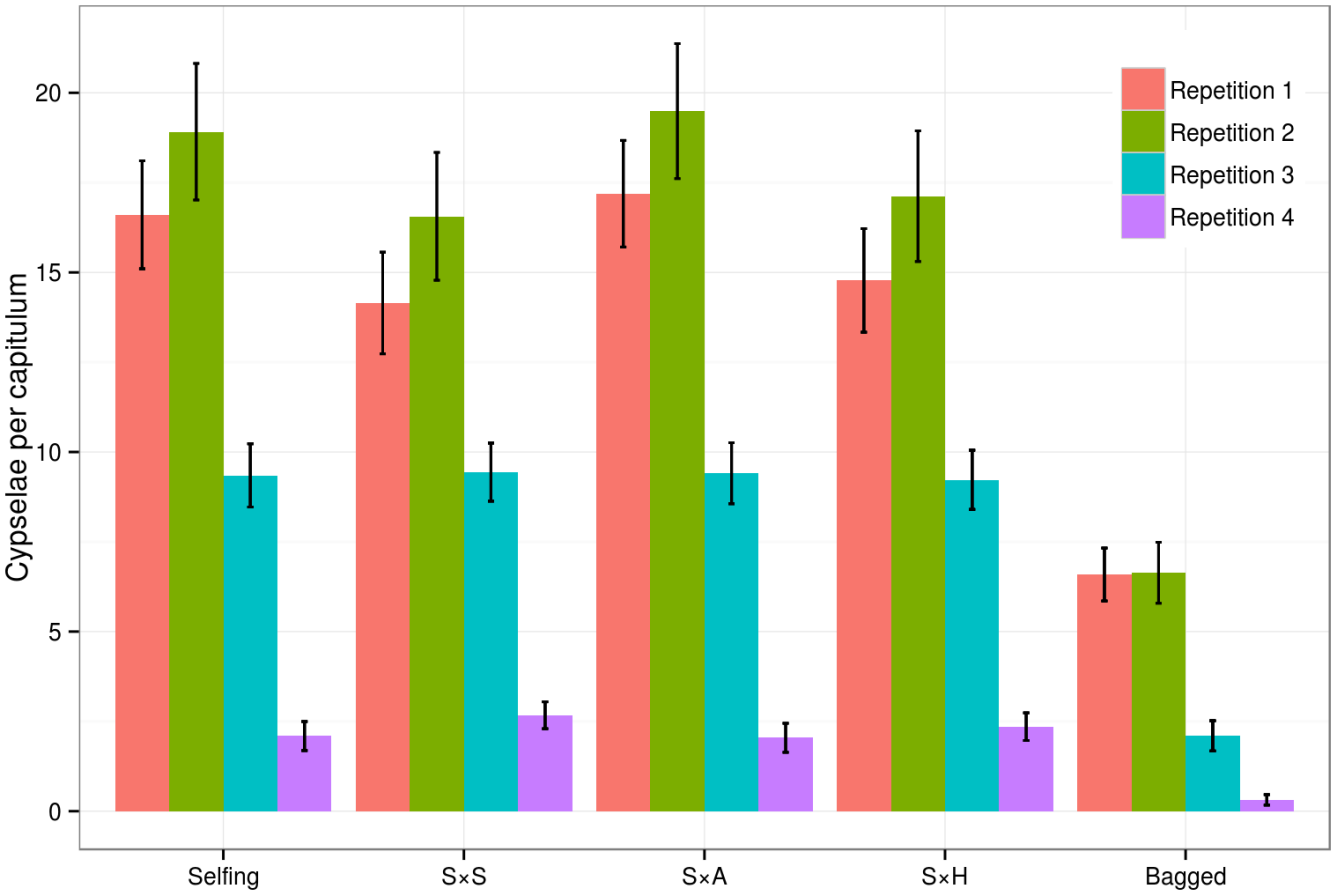
Predicted values for the tetraploid *Centaurea seridis* by the Zero inflated Poisson model. Colors represent the repetitions; red: first repetition; green: second; blue: third and purple: fourth. Selfing: self-pollination; S×S: cross-pollination between individuals of *C*. *seridis* (intraploidy); S×A: coss-pollination between mother tetraploid *C*. *seridis* and father diploid *C*. *aspera*; S×H: coss-pollination between mother tetraploid *C*. *seridis* and father triploid *C*. × *subdecurrens*; bagged: bagged treatment without brushing. Error bars indicate the standard error for the Poisson model estimates.

When using the ovules from *C*. *seridis* and the pollen from *C*. *aspera* (S×A) and *C*. × *subdecurrens* (S×H), the interploidy pollinations resulted in 176 cypselae in 16 capitula, and 171 cypselae in 17 capitula, respectively. These results are similar to those obtained in the *C*. *seridis* self- and intraploidy pollinations, and no significant differences were observed among these treatments (Kruskal-Wallis, S2 Table and Fig 3). There were only a few cypselae with small embryos for S×A and S×H (2 and 3, respectively), and there were 34 and 33 depredated cypselae, respectively. Finally, it was not possible to fertilize the ovules of *C*. × *subdecurrens* by either the *C*. *aspera* (H×A) or the *C*. *seridis* (H×S) pollen, and no cypselae were detected in 31 capitula with interploidy pollinations.

### Seasonal influence

Four repetitions of all the treatments were performed from the start of June to the start of July. The seasonality effect was analyzed among all the treatments and taxa, and significant differences between repetitions were observed (Kruskal-Wallis, S3 Table and S2 Fig). Nonparametric models showed that the number of cypselae per capitulum lowered over time, although the relationships among treatments remained largely unchanged (Figs 2 and 3).

### Progeny ploidy level

As expected, the intact cypselae originating from the self-pollinations and intraploidy pollinations of the *C*. *aspera* individuals were diploid, and those of *C*. *seridis* were tetraploid (Table 1). However, one small embryo produced by the *C*. *aspera* intraploidy pollination was triploid, as was one small embryo obtained in a *C*. *seridis* self-pollination. One small aneuploid embryo was also obtained in the *C*. *seridis* intraploidy pollination.

The interploidy pollinations between *C*. *aspera* and *C*. *seridis* gave different ploidy results, depending on which taxon acted as the male and female parent. When *C*. *aspera* acted as the female parent (A×S), the resulting nine intact cypselae were eight triploids and one aneuploid. When *C*. *seridis* acted as the female parent (S×A), 140 progeny intact cypselae and two small embryos were all tetraploid, which suggests that these cypselae are effectively produced by selfing and not by *C*. *aspera* pollen. In fact similar results were obtained when infertile *C*. × *subdecurrens* was used as the male parent, and 135 tetraploid intact cypselae, two tetraploid small embryos and one triploid small embryo were obtained, while 50 tetraploid intact cypselae, three tetraploid small embryos and one triploid small embryo were obtained in the bagged treatment (Table 1).

### Progeny germination

All the intact cypselae germinated in only one day after removing the cypsela pericarp. Therefore if only intact cypselae were taken into account (without pericarp and with cut cotyledons), no significant differences in the germination and germination rate were detected among the taxa or pollination treatments. This result suggests physical exogenous dormancy, which is regulated exclusively by the pericarp. In contrast, all the small embryos except one (a A×S triploid small embryo) did not germinate.

## Discussion

### Phenological differentiation among taxa

Despite there being only 2 to 3 weeks difference in the start of anthesis, the mean overlap in flowering between diploid *C*. *aspera* and tetraploid *C*. *seridis* was 50%. This result was similar to that found in *Chamerion angustifolium* (L.) Holub, where the overlap between diploids and tetraploids was 51%, despite their similar flowering duration and after only 1 week of asynchrony [24]. This demonstrates that flowering phenology is a significant prezygotic barrier that increases assortative mating [32].

In all the treatments, the number of cypselae per capitulum lowered from early to late flowering. This was also observed in *Centaurea corymbosa* Pourr., and was probably because the resource availability for ovule production decreased as leaf rosettes dried and as developing seeds from earlier flowers presented increasing demand [33].

### Fertile *vs*. sterile pollen and eggs from triploid *C*. × *subdecurrens*

A strong triploid block exists in the majority of angiosperms, which prevents hybridization between diploids and tetraploids [34]. Nevertheless, while the triploid block can be strong, it is not absolute as triploids are generally found naturally, even if they are produced at very low frequencies or obtained in experimental crosses [35]. In these cases, triploids may be partially fertile and can act as a triploid bridge, which could lead to some gene flow between diploids and their related tetraploids [36, 37].

In the studied contact zone, natural sterile triploid hybrids are frequent [14]. According to our results, the pollen and eggs from triploids in the forced crosses were totally sterile. Thus, no triploid bridge took place, which is in agreement with previous studies that used molecular data and supported a very low or no gene flow [14, 15].

### Allogamy *vs*. autogamy in diploids and tetraploids

The diploid *C*. *aspera* can be considered self-incompatible. Self-pollination treatment yielded only three cypselae with well-developed embryos. These three embryos were diploid, and the facts that they were produced in only one capitulum out of 16 and that the same individual (444N 66W) had a bagged and an A×H treatments with 0 seeds suggest pollen contamination (S1 Data). Nevertheless a very small percentage of self-compatibility or pseudo-self-compatibility cannot be totally ruled out [38].

A breakdown of self-incompatibility (SI) may benefit polyploids because it can increase their fitness by rendering the presence of mating partners of the same ploidy level unnecessary [39]. Specifically, there is evidence for a tendency of a breakdown of SI in polyploids with gametophytic SI systems [40, 41]. However, Asteraceae possess a sporophytic SI system and consistently retain SI [42, 43]. Despite more than one third of Asteraceae species exhibiting a partial or complete breakdown of SI, polyploidization does not seem to be the cause [44].

Accordingly, in the genus *Centaurea*, all the studied diploid and tetraploid related taxa are highly self-incompatible [5, 6]. Here tetraploid *C*. *seridis* showed high selfing rates, at least in forced pollinations. To our knowledge, this is the first report of autogamous allotetraploids that originated from one allogamous diploid progenitor in *Centaurea*. Some diploid *Centaurea* species show partial self-compatibility, but not related to polyploidization processes [38, 45]. Although experimental crossings suggest a high selfing rate in *C*. *seridis*, this could not be confirmed in nature, as no genetic analysis of both parental and progeny was performed [46]. The bagged treatment proved that *C*. *seridis* was able to automatically self-pollinate (including geitonogamy), although with a frequency lower than self-pollination treatment (with brushing). Some of these cypselae may be due to apomixis, which was not tested, but which is very unlikely in *Centaurea* [15]. Brushing probably promotes arrival of pollen to the stigma to improve fertilization and seed formation, and thus the lack of brushing might explain the smaller number of cypselae in the bagged treatment. However, a potential reduction of the effective population size in *C*. *seridis* is expected even with variable selfing rates, leading to genetic drift. Genetic drift may partly explain the low levels of genetic diversity within populations observed in *C*. *seridis* when compared to its only known diploid progenitor *C*. *aspera* [14, 15]. Rare polyploidization events and bottleneck effects in *C*. *seridis* could also explain its lower genetic diversity [15, 47]. In fact, in other Asteraceae taxa, bottleneck effects may have caused the breakdown of SI, as a result of a predicted reduction in the number of S-alleles and compatible mates, especially in highly colonizing, annual or short-lived perennials [44, 48–50]. However, loss of SI is reversible in Asteraceae [44], and the re-establishment of SI systems may be facilitated when the number of mates rises and the selection against rare taxa decreases. According to our results, brushing increased seed set in *C*. *seridis*, which highlights the important but not essential role of pollinators despite self-compatibility.

### Seed set intra- *vs*. inter-specific and ploidy level of offspring

The studied contact zone met the general rule by which inter-ploidy crosses yield less seeds than intra-ploidy crosses, save S×A because of selfing in *C*. *seridis* [5, 8, 10, 43, 51].

The offspring from the intraploidy level crosses in *C*. *aspera* (A×A) included mainly diploids (98.4%), and one triploid (1.6%) that failed to germinate. This triploid may have originated through the formation of one unreduced gamete by a diploid parental. This result suggests a low production rate of unreduced gametes. A low or null frequency of unreduced gametes has already been found in other *Centaurea* species, such as *C*. *jacea* L., *C*. *phrygia* L. and *C*. *pseudophrygia* C.A. Mey. [5, 6, 8].

Similarly, all the offspring were tetraploid in the S×S treatment in *C*. *seridis*, except for an aneuploid embryo (0.2%), which did not germinate either. In the synthesized allotetraploids, the genetic changes caused by homologous chromosome rearrangement were found to be common, and led to the occurrence of multivalents and univalents at meiosis, which resulted in the production of gametes with unbalanced chromosomal composition [52]. Thus aneuploid gametes can be produced by polyploid meiosis, although their frequency may vary among species [53].

The ploidy level of the offspring from the interploidy crosses was highly asymmetrical. Diploid mothers always yielded triploid individuals when pollinated by tetraploids (A×S), while tetraploid mothers only yielded tetraploid individuals when pollinated by diploids (S×A). In the A×S crosses, we observed no diploid progeny, not even when flowers were not emasculated, which supports the absence of mentor effects, a frequent mechanism that has been described in both allogamous diploids and tetraploids [6, 8, 36, 51, 54]. In A×S crosses a high percentage of aberrations was also produced, probably due to irregularities in the embryo formation process. The fact that no well developed triploids were found in the S×A crosses supports the autogamy of *C*. *seridis*, but also suggests a mentor effect. In *Chamerion angustifolium*, Husband *et al*. [55] and Baldwin and Husband [56] found higher pollen siring rates in mixed pollen loads in established tetraploids than in diploids. Unilateral tetraploid pollen priority has also been observed in *Hieracium echioides* Lumn. [36].

## Conclusions

*Centaurea aspera* is a species with broad populations from inland to the coast, while *C*. *seridis* has a smaller distribution area, located exclusively on the coast and largely overlapped by *C*. *aspera*. Assortative mating through prezygotic isolation was much higher in tetraploid *C*. *seridis* than in diploid *C*. *aspera*. Here we demonstrated that several mechanisms related with floral biology acted in tetraploids, such as a flowering phenological shift, breakdown in self-incompatibility and siring in the production of sterile triploid hybrids. These mechanisms may diminish or suppress the crosses between diploid father and tetraploid mother in the contact zones, thus helping tetraploids to coexist in sympatry with diploids, whereas they can reduce genetic diversity and lead to a strong genetic structure in tetraploids. Whether these mechanisms can influence pollinator preferences or microspatial cytotype segregation through long or short dispersals of cypselae, especially triploid seeds that are produced only by diploid mothers, remains to be resolved. Minor ecological differences between taxa have been suggested in the contact zones where they coexist [14], although microspatial distribution of the three taxa has not yet been studied.

## Supporting Information

S1 Data Number of cypselae for each treated capitula. (XLS)

S1 Fig Histogram, density plot (black line), normal distribution (red line) and Shapiro-Wilk normality test for the number of cypselae per capitulum for each pollination treatment and taxon. (TIFF)

S2 Fig Box and whisker plots among *Centaurea aspera* pollination treatments; *C*. *seridis* pollination treatments; intraploidy pollination in *C*. *aspera* (A×A) repetitions; and intraploidy pollinations in *C*. *seridis* (S×S) repetitions. (TIF)

S3 Fig Photographs of the three studied taxa. From left to right: *C*. *aspera* (diploid), *C*. × *subdecurrens* (triploid) and *C*. *seridis* (tetraploid); habitat (upper); location: study area (red triangle), Albufera lake (blue), Albufera Natural Park (green), Valencia province (grey).
(TIF)

S1 Table Repetition data: bagging date, first hand-pollination, second hand-pollination and number of cypselae for each repetition. (PDF)

S2 Table Kruskal-Wallis and Dunn's tests for the number of cypselae among pollination treatments. (PDF)

S3 Table Kruskal-Wallis and Dunn's tests for the number of cypselae among repetitions. (PDF)
