# Supplementary figures and images for: Allogamy-Autogamy Switch Enhance Assortative Mating in the Allotetraploid Centaurea seridis L. Coexisting with the Diploid Centaurea aspera L. and Triggers the Asymmetrical Formation of Triploid Hybrids
Source: PLoS One. 2015 Oct 15;10(10):e0140465. doi: 10.1371/journal.pone.0140465 (PMC4607450; doi:10.1371/journal.pone.0140465)

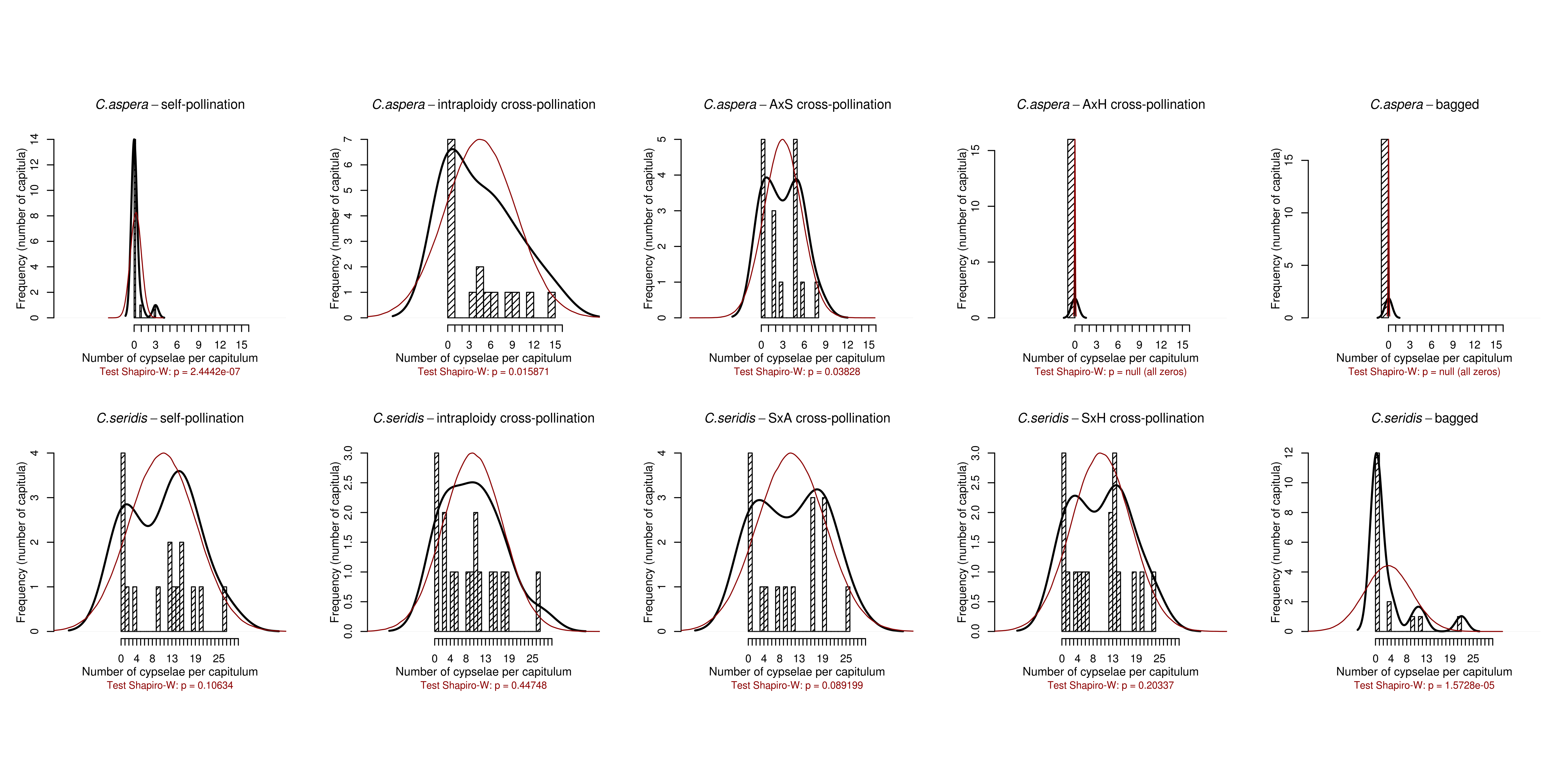

Supplement: S1 Fig — (TIFF) [file pone.0140465.s002.tiff]

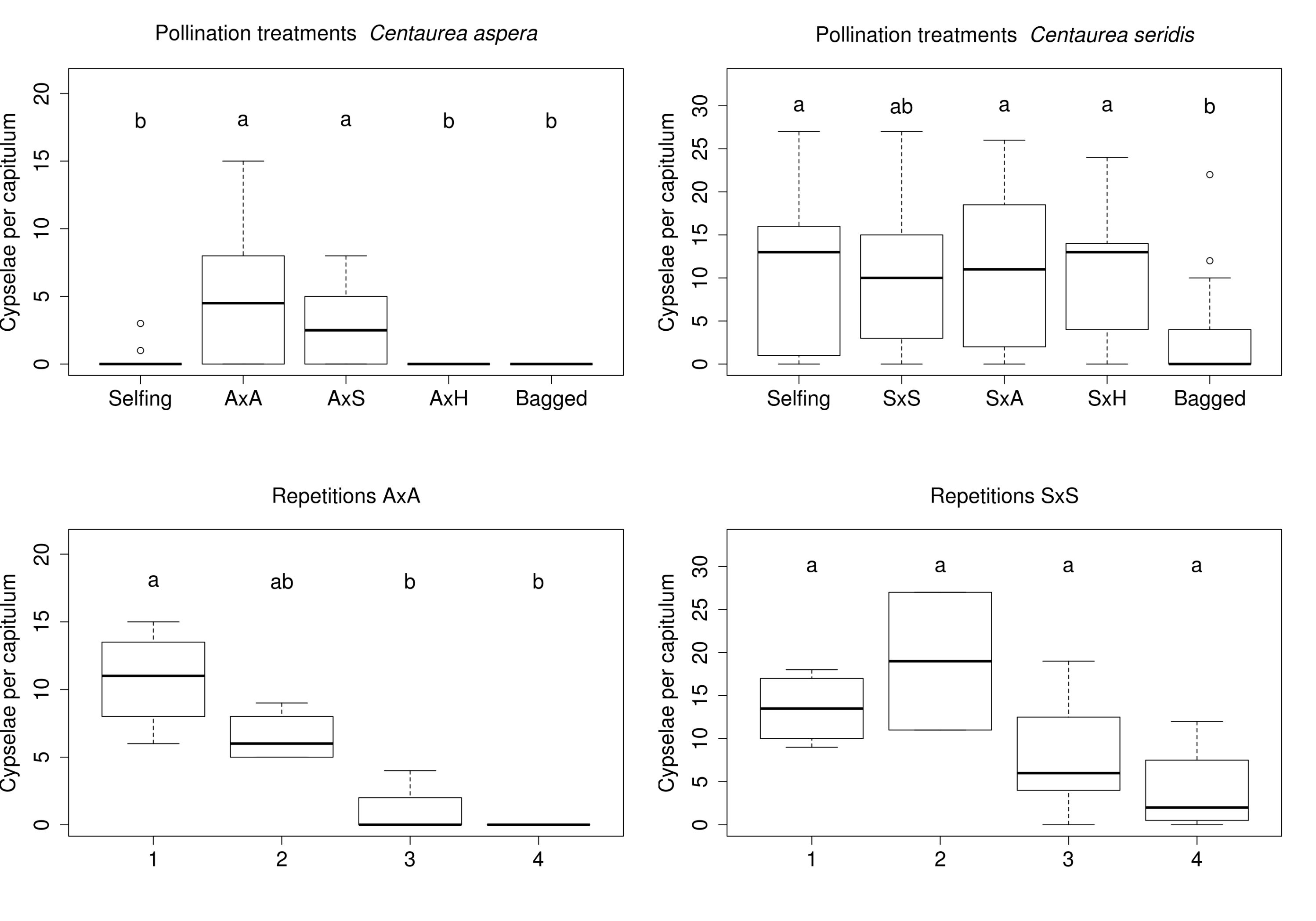

Supplement: S2 Fig — (TIF) [file pone.0140465.s003.tif]

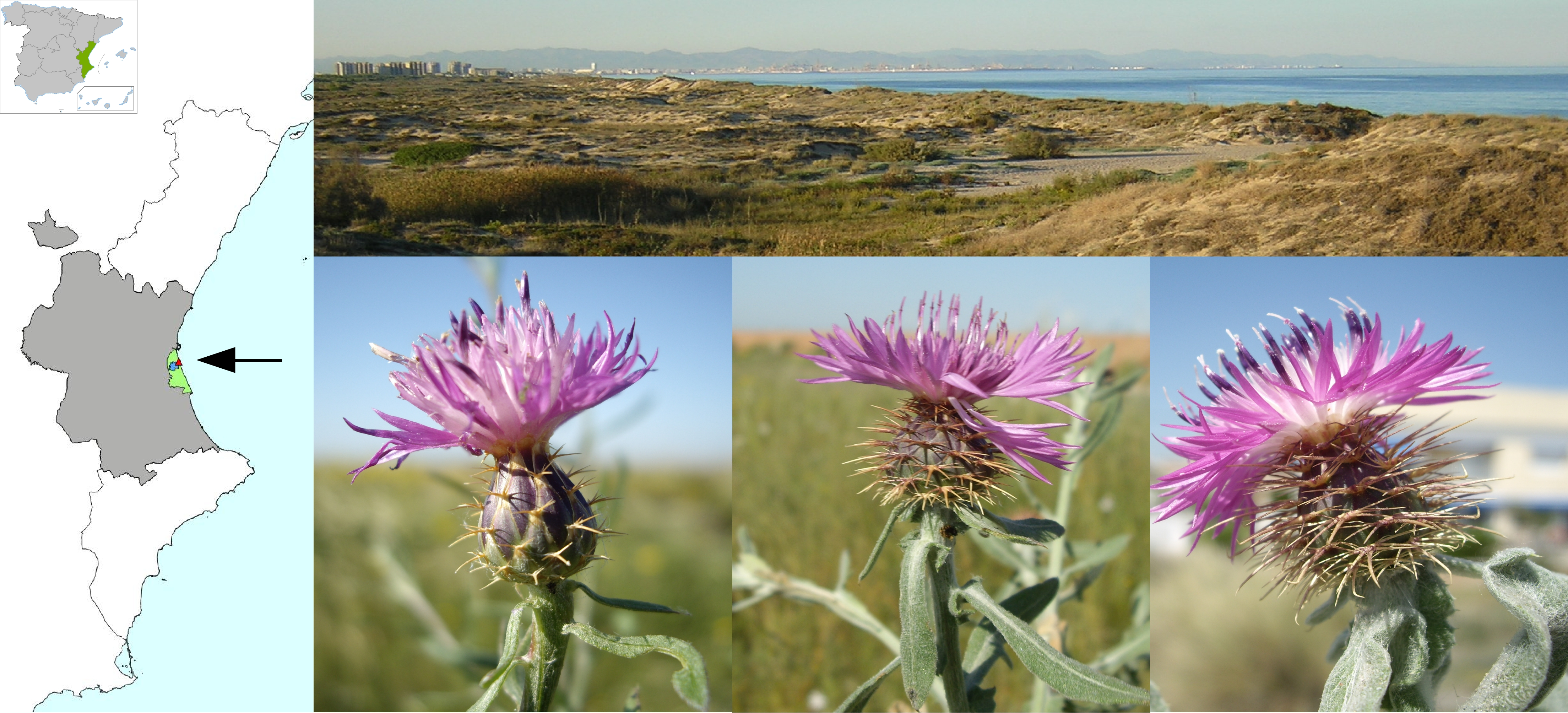

Supplement: S3 Fig — From left to right: C. aspera (diploid), C. × subdecurrens (triploid) and C. seridis (tetraploid); habitat (upper); location: study area (red triangle), Albufera lake (blue), Albufera Natural Park (green), Valencia province (grey). (TIF) [file pone.0140465.s004.tif]
