# Supplementary material for: Allogamy-Autogamy Switch Enhance Assortative Mating in the Allotetraploid Centaurea seridis L. Coexisting with the Diploid Centaurea aspera L. and Triggers the Asymmetrical Formation of Triploid Hybrids
Source: PLoS One. 2015 Oct 15;10(10):e0140465. doi: 10.1371/journal.pone.0140465 (PMC4607450; doi:10.1371/journal.pone.0140465)
Supplement: S1 Table — (PDF) [file pone.0140465.s005.pdf]

Repetition	Bagging date	First hand-pollination	Second hand-pollination	Number of capitula			
				Total	*C. aspera*	*C. seridis*	*C. x subdecurrens*
Repetition 1	17-Jun-13	19-Jun-13	20-Jun-13	56	20	20	16
Repetition 2	24-Jun-13	27-Jun-13	28-Jun-13	47	20	10	17
Repetition 3	26-Jun-13	29-Jun-13	30-Jun-13	80	21	34	25
Repetition 4	5-Jul-13	8-Jul-13	9-Jul-13	59	20	19	20
Total				242	81	83	78
