# Supplementary material for: Allogamy-Autogamy Switch Enhance Assortative Mating in the Allotetraploid Centaurea seridis L. Coexisting with the Diploid Centaurea aspera L. and Triggers the Asymmetrical Formation of Triploid Hybrids
Source: PLoS One. 2015 Oct 15;10(10):e0140465. doi: 10.1371/journal.pone.0140465 (PMC4607450; doi:10.1371/journal.pone.0140465)
Supplement: S2 Table — (PDF) [file pone.0140465.s006.pdf]

Maternal taxon	chi-squared	df	p-value	mean	+-	se	Dunn's	mean	+-	se	Dunn's	mean	+-	se	Dunn's	mean	+-	se	Dunn's	mean	+-	se	Dunn's
Treatments				Selfing				A×A				A×S				A×H				Bagged			
C. aspera	34.8297	4	0.0000005	0.25	+-	0.19	b	4.56	+-	1.24	a	3.00	+-	0.68	a	0.00	+-	0.00	b	0.00	+-	0.00	b
Treatments				Selfing				S×S				S×A				S×H				Bagged			
C. seridis	11.4256	4	0.0221747	10.63	+-	2.16	a	9.76	+-	1.84	ab	11.00	+-	2.19	a	10.06	+-	1.86	a	3.24	+-	1.47	b
