# Supplementary material for: Allogamy-Autogamy Switch Enhance Assortative Mating in the Allotetraploid Centaurea seridis L. Coexisting with the Diploid Centaurea aspera L. and Triggers the Asymmetrical Formation of Triploid Hybrids
Source: PLoS One. 2015 Oct 15;10(10):e0140465. doi: 10.1371/journal.pone.0140465 (PMC4607450; doi:10.1371/journal.pone.0140465)
Supplement: S3 Table — (PDF) [file pone.0140465.s007.pdf]

Species	Treatments	chi-squared	df	p-value	Repetition 1				Repetition 2				Repetition 3				Repetition 4			
					mean	+-	se	Dunn's	mean	+-	se	Dunn's	mean	+-	se	Dunn's	mean	+-	se	Dunn's
C. aspera	Selfing	2.1500	3	0.5419	0.75	+-	0.75	a	0.25	+-	0.25	a	0.00	+-	0.00	a	0.00	+-	0.00	a
	A×A	13.2905	3	0.0040	10.75	+-	1.89	a	6.50	+-	0.96	ab	1.00	+-	1.00	b	0.00	+-	0.00	b
	A×S	8.1368	3	0.0433	4.50	+-	1.50	ab	5.00	+-	0.00	a	2.00	+-	1.22	ab	0.50	+-	0.50	b
	A×H	-	3	-	0.00	+-	0.00	-	0.00	+-	0.00	-	0.00	+-	0.00	-	0.00	+-	0.00	-
	Bagged	-	3	-	0.00	+-	0.00	-	0.00	+-	0.00	-	0.00	+-	0.00	-	0.00	+-	0.00	-
C. seridis	Selfing	9.9607	3	0.0189	20.00	+-	2.89	a	7.50	+-	7.50	ab	12.17	+-	1.87	ab	0.50	+-	0.50	b
	S×S	5.4939	3	0.1390	13.50	+-	2.10	a	19.00	+-	8.00	a	8.29	+-	2.56	a	4.00	+-	2.74	a
	S×A	6.9740	3	0.0727	14.25	+-	2.66	ab	23.00	+-	3.00	a	9.14	+-	3.43	ab	3.00	+-	1.53	b
	S×H	7.6955	3	0.0527	13.00	+-	2.45	ab	19.00	+-	5.00	a	10.57	+-	2.79	ab	1.75	+-	1.18	b
	Bagged	8.1857	3	0.0423	9.50	+-	4.86	a	7.00	+-	3.00	a	0.29	+-	0.18	a	0.25	+-	0.25	a
